# Draft genome of the emerging pathogen, *Kocuria marina,* isolated from a wild urban rat

**DOI:** 10.1590/0074-02760170132

**Published:** 2017-12

**Authors:** Shih Keng Loong, Kim-Kee Tan, Nurhafiza Zainal, Wai Hong Phoon, Siti Nursheena Mohd Zain, Sazaly AbuBakar

**Affiliations:** 1 University of Malaya, Faculty of Medicine, Tropical Infectious Diseases Research & Education Centre, Kuala Lumpur, Malaysia; 2 University of Malaya, Faculty of Medicine, Department of Medical Microbiology, Kuala Lumpur, Malaysia; 3 University of Malaya, Faculty of Science, Institute of Biological Sciences, Kuala Lumpur, Malaysia

**Keywords:** infectious disease, Kocuria, zoonosis, Malaysia, Rattus rattus

## Abstract

*Kocuria marina* has recently emerged as a cause for catheter-related bloodstream infections in patients with underlying health complications. One K. *marina* strain was recently isolated from the lung tissues of a wild urban rat *(Rattus rattus diardii)* caught during rodent surveillance. Here, we present the draft genome of the first K. *marina* animal isolate, K. *marina* TRE150902.

*Kocuria marina* is a Gram positive cocci, isolated initially from marine sediment (Kim et al. 2004) and shares identical morphology to *Staphylococci* and *Micrococci* (Kandi et al. 2016). Although predominantly found in the environment and, as normal flora on the skin and oropharynx of mammals, clinical cases are increasingly described (Purty et al. 2013), signifying the pathogenic potential of this bacterial species. Clinical cases reported in the literature involved the elderly and the young with underlying health complications and were mostly associated with catheter usage (Lee et al. 2009, Lai et al. 2011, Brandle et al. 2014, Horiuchi et al. 2015, Mori et al. 2017). *K. marina* was also recently isolated from the lung tissues of a wild urban rat (Loong et al. 2016), potentially adding a zoonotic dimension into the transmission cycle.

Here, we present the draft genome of *K. marina* TRE150902 isolated from rat lung tissues. Strain TRE150902 was susceptible to common antibiotics and was confirmed as K. *marina* by 16S rDNA sequencing and other phenotypic tests (Loong et al. 2016). Strain TRE150902 was cultured overnight in brain-heart infusion broth under aerobic conditions at 37°C and, the resulting bacterial culture was used for transmission electron microscopy (Tan & Suresh 2006) and genome sequencing.

Bacterial cells from late-exponential phase were fixed overnight at 4°C with 4% (vol/vol) glutaraldehyde and 0.1M sodium cacodylate buffer. The bacterial cells were subsequently washed with sterile water and dehydrated using increasing concentrations of ethanol. Following that, bacterial cells were embedded overnight in epoxy resin and then sliced into ultrathin sections using a diamond knife (Diatome, USA). Sections were mounted on a mesh copper grid (Ted Pella, USA), stained with uranyl acetate and then imaged using a HT7700 Transmission Electron Microscope (Hitachi, Japan).

Whole genome sequencing of K. *marina* TRE150902 was performed as previously described (Tan et al. 2015, Loong et al. 2017) with minor modifications. Genome library preparation was performed using Ion Xpress Plus Fragment Library Kit (Thermo Fisher Scientific, USA) and genome libraries corresponded to 200bp were prepared using E-Gel SizeSelect Agarose Gel, 2% (Thermo Fisher Scientific, USA). The sequencing template was prepared using Ion PGM Hi-Q OT2 Kit (Thermo Fisher Scientific, USA) according to manufacturer's protocol. Amplified Ion Sphere Particles were enriched using Ion PGM Enrichment beads (Thermo Fisher Scientific, USA) and genome sequencing was undertaken using the Ion Torrent PGM sequencer (Life Technologies, USA) using Ion PGM Hi-Q sequencing kit. The Ion Torrent reads were assembled *de novo* using SPAdes v3.1.0 (Bankevich et al. 2012) and the assembled contigs were functionally annotated with Rapid Annotation using Subsystem Technology (RAST) (Aziz et al. 2008).

Transmission electron microscopy revealed that the morphological characteristics of *K. marina* resembled *Staphylococcus aureus* (Touhami et al. 2004), showing observable growth and division by the formation of septum (Figure). The resulting draft genome of *K. marina* TRE150902 was 2,856,751 bp in length, comprising of 54 contigs and N_50_ of 98,614. The GC content of the draft genome was approximately 68.9% and a total 2607 protein-coding genes with 50 RNAs were predicted using RAST. These results were summarised in Table.

**Figure:**
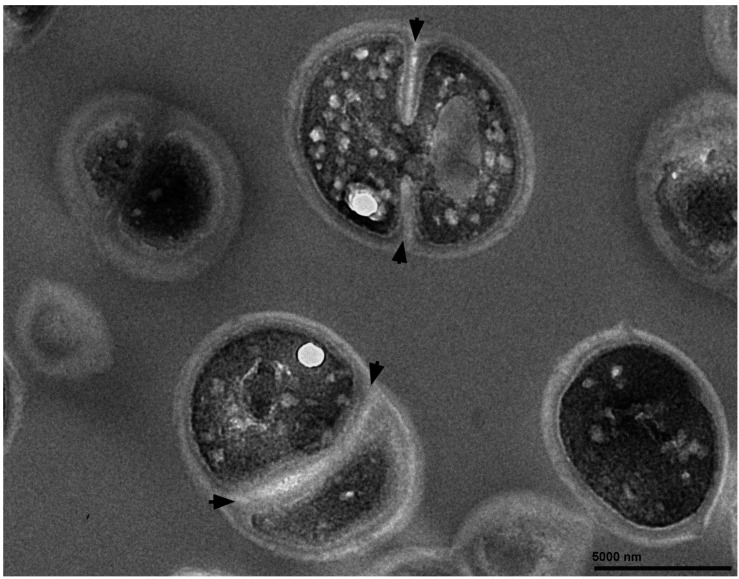
Morphological characteristics of *Kocuria marina* TRE150902 as shown by transmission electron microscopy at 12,000 times magnification. Arrows show the formation of septa.

**TABLE:** Overview of *Kocuria marina* TRE150902 genome assembly

Attribute	Chromosome
Genome size (bp)	2,856,751
GC content (%)	68.9
Contigs	54
Open reading frames	2697
RNA	50

Genome annotation in the RAST server uncovered the presence of genes coding for enzymes involved in invasion and intracellular resistance, which include quinolinate synthetase (EC 2.5.1.72), quinolinate phosphoribosyltransferase [decarboxylating] (EC 2.4.2.19), L-aspartate oxidase (EC 1.4.3.16), inner membrane protein translocase component YidC, protein YidD and RNA-binding protein Jag. In addition, genes responsible for resistance to toxic compounds (mercuric ion reductase (EC 1.16.1.1), copper resistance protein CopC, cobalt-zinc-cadmium resistance protein CzcD and arsenical-resistance protein ACR3) were also annotated, potentially aiding K. *marina* TRE150902 proliferation in various environments (Purty et al. 2013), outside the host.

The potential zoonotic transmission of *K. marina* which otherwise known to be transmitted only from the environment to humans would undoubtedly confound disease and risk management of the bacteria. These highlight the emerging complexity of interactions between human, animal, environmental factors and new bacterial pathogens. The draft genome of *K. marina* TRE150902 will aid genomic analyses and comparison with other clinical and environmental strains to elucidate the potential diversity between them. The draft genome sequences generated in this study are available from the European Nucleotide Archive under the study number PRJEB19997.
